# Supplementary material for: Upper extremity deep vein thrombosis in COVID-19: Incidence and correlated risk factors in a cohort of non-ICU patients
Source: PLoS One. 2022 Jan 12;17(1):e0262522. doi: 10.1371/journal.pone.0262522 (PMC8754283; doi:10.1371/journal.pone.0262522)
Supplement: S1 Appendix — (DOCX) [file pone.0262522.s001.docx]

| **Suppl table 1** | LEDVT (27) | No-LEDVT (230) | p |
| --- | --- | --- | --- |
| Age, mean±SD | 73,5±10,72 | 69,73±12,37 | n.s. |
| Male, n (%) | 20 (74,1%) | 164 (71,3%) | n.s. |
| Days of hospitalization, mean±SD | 21,14±12,85 | 19,14±12,19 | n.s. |
| Disease severity: |  |  |  |
| - PaO2/Fio2, mean±SD | 211,19±79,30 | 251,36±82,94 | <0,05 |
| - ARDS, n (%) | 16 (59,3%) | 104 (45,2%) | n.s. |
| - Associated Pneumonia, n (%) | 23 (85,2%) | 185 (84,3%) | n.s. |
| Patients requiring CPAP, n (%) | 21 (77,8%) | 143 (62,2%) | n.s. |
| - Days of ventilation, mean±SD | 11,48±6,65 | 10,38±5,94 | n.s. |
| Associated comorbidities: |  |  |  |
| - Heart Failure, n (%) | 3 (11,1%) | 36 (15,6%) | n.s. |
| - COPD, n (%) | 1 (3,7%) | 29 (12,6%) | n.s. |
| - Diabetes, n (%) | 3 (11,5%) | 51 (22,2%) | n.s. |
| - Hypertension, n (%) | 14 (53,8%) | 127 (55,2%) | n.s. |
| - History of Stroke, n (%) | 0 (0%) | 9 (3,9%) | n.s. |
| - Dementia, n (%) | 2 (7,4%) | 22 (9,6%) | n.s. |
| - Body mass index >30 kg/m2, n (%) | 3 (11,1%) | 33 (14,8%) | n.s. |
| - Cancer, n (%) | 1 (3,7%) | 8 (3,5%) | n.s. |
| - Thrombophilia, n (%) | 6 (22,2%) | 16 (6,9%) | <0,01 |
| Blood test analysis: |  |  |  |
| - Creatinine, mean±SD | 1,13±0,56 | 1,03±0,62 | n.s |
| - Hemoglobin, mean±SD | 12,90±1,80 | 13,40±1,74 | n.s |
| - White cells count, mean±SD | 9,21±4,81 | 8,33±6,07 | n.s. |
| - Platelet count, mean±SD | 259703,7±102374,1 | 233469,0±105585,8 | n.s |
| - Reattive C protein, mean±SD | 15,57±10,95 | 10,56±17.52 | n.s |
| - D-dimer, mean±SD | 7250,48±8743,32 | 2736,12±7187,47 | <0,01 |
| - D-dimer > age adjusted range, n (%) | 25 (92,6%) | 71 (30,9%) | <0,0001 |
| Deaths, n (%) | 7 (25,9%) | 46 (20,1%) | n.s. |

| **Suppl table 2** | DVT (45) | No-DVT (212) | p |
| --- | --- | --- | --- |
| Age, mean±SD | 73,26±10,24 | 69,47±12,55 | n.s. |
| Male, n (%) | 34 (75,6%) | 150 (70,7%) | n.s. |
| Days of hospitalization, mean±SD | 22,22±13,35 | 18,75±11,95 | n.s. |
| Disease severity: |  |  |  |
| - PaO2/Fio2, mean±SD | 216,88±77,56 | 253,60±83,29 | <0,01 |
| - ARDS, n (%) | 30 (66,7%) | 90 (42,4%) | <0,01 |
| - Associated Pneumonia, n (%) | 41 (91,1%) | 167 (78,8%) | n.s. |
| Patients requiring CPAP, n (%) | 37 (82,2%) | 127 (59,9%) | <0,01 |
| - Days of ventilation, mean±SD | 11,81±6,19 | 10,15±5,95 | n.s. |
| Associated comorbidities: |  |  |  |
| - Heart Failure, n (%) | 7 (15,6%) | 32 (15,1%) | n.s. |
| - COPD, n (%) | 2 (4,4%) | 28 (13,2%) | n.s. |
| - Diabetes, n (%) | 4 (9,3%) | 50 (23,6%) | <0,05 |
| - Hypertension, n (%) | 23 (53,4%) | 118 (55,7%) | n.s. |
| - History of Stroke, n (%) | 0 (0%) | 9 (4,2%) | n.s. |
| - Dementia, n (%) | 3 (6,7%) | 21 (9,9%) | n.s. |
| - Body mass index >30 kg/m2, n (%) | 5 (11,1%) | 32 (15,1%) | n.s. |
| - Cancer, n (%) | 1 (2,2%) | 8 (3,8%) | n.s. |
| - Thrombophilia, n (%) | 8 (17,8%) | 14 (6,6%) | <0,05 |
| Blood test analysis: |  |  |  |
| - Creatinine, mean±SD | 1,05±0,49 | 1,04±0,65 | n.s |
| - Hemoglobin, mean±SD | 13,30±1,69 | 13,36±1,77 | n.s |
| - White cells count, mean±SD | 9,58±4,53 | 8,18±6,19 | n.s. |
| - Platelet count, mean±SD | 258181,8±101049,0 | 231655,0±105903,3 | n.s |
| - Reattive C protein, mean±SD | 13,92±9,06 | 10,50±18.20 | n.s |
| - D-dimer, mean±SD | 7671,65±10102,49 | 2255,6±6394,91 | <0,0001 |
| - D-dimer > age adjusted range, n (%) | 39 (86,7%) | 57 (26,9%) | <0,0001 |
| Deaths, n (%) | 15 (33,3%) | 38 (17,9%) | <0,05 |

| **Suppl table 3** | Dead (53) | Non-dead (204) | p |
| --- | --- | --- | --- |
| Age, mean±SD | 78,21±9,08 | 68,04±12,10 | <0,0001 |
| Male, n (%) | 41 (77,4%) | 143 (70,1%) | n.s. |
| Days of hospitalization, mean±SD | 11,73±9,19 | 21,34±12,19 | <0,001 |
| Disease severity: |  |  |  |
| - PaO2/Fio2, mean±SD | 210,67±97,77 | 256,85±76,33 | <0,0001 |
| - ARDS, n (%) | 32 (60,4%) | 88 (43,1%) | <0,05 |
| - Associated Pneumonia, n (%) | 44 (83,0%) | 164 (80,4%) | n.s. |
| Patients requiring CPAP, n (%) | 41 (77,3%) | 123 (60,3%) | <0,05 |
| - Days of ventilation, mean±SD | 7,63±5,18 | 11,49±6,00 | <0,001 |
| Associated comorbidities: |  |  |  |
| - Heart Failure, n (%) | 14 (26,4%) | 25 (12,2%) | <0,05 |
| - COPD, n (%) | 11 (20,7%) | 19 (9,3%) | <0,05 |
| - Diabetes, n (%) | 14 (26,9%) | 40 (19,6%) | n.s. |
| - Hypertension, n (%) | 36 (67,9%) | 105 (51,5%) | n.s. |
| - History of Stroke, n (%) | 3 (5,7%) | 6 (2,9%) | n.s. |
| - Dementia, n (%) | 15 (28,3%) | 9 (4,4%) | <0,0001 |
| - Body mass index >30 kg/m2, n (%) | 6 (11,3%) | 31 (15,2%) | n.s. |
| - Cancer, n (%) | 2 (3,8%) | 7 (3,4%) | n.s. |
| - Thrombophilia, n (%) | 4 (7,5%) | 18 (8,9%) | n.s. |
| Blood test analysis: |  |  |  |
| - Creatinine, mean±SD | 1,19±0,49 | 1,01±0,64 | n.s |
| - Hemoglobin, mean±SD | 13,50±1,54 | 13,31±1,80 | n.s |
| - White cells count, mean±SD | 9,09±5,01 | 8,26±6,17 | n.s. |
| - Platelet count, mean±SD | 203153,8±76094,73 | 244835,8 ±110235,8 | n.s |
| - Reattive C protein, mean±SD | 11,01±8,71 | 11,12±18,60 | n.s |
| - D-dimer, mean±SD | 3791,32±7839,23 | 3121,74±7430,47 | n.s. |
| - D-dimer > age adjusted range, n (%) | 25 (44,17%) | 71 (34,8%) | n.s. |
| UEDVT | 13 (24,5%) | 15 (7,3%) | <0,0001 |
| LEDVT | 7 (13,2%) | 20 (9,8%) | n.s. |
| DVT | 15 (28,3%) | 30 (14,7%) | <0,05 |
| **Suppl. Table 3:** Comparison between dead and survivors. | | | |
|  |  |  |  |
